# Morphometric magnetic resonance imaging and genetic testing in cerebellar abiotrophy in Arabian horses

**DOI:** 10.1186/1746-6148-9-105

**Published:** 2013-05-23

**Authors:** Jessika MV Cavalleri, Julia Metzger, Maren Hellige, Virginie Lampe, Kathrin Stuckenschneider, Andrea Tipold, Andreas Beineke, Kathrin Becker, Ottmar Distl, Karsten Feige

**Affiliations:** 1 Clinic for Horses, University of Veterinary Medicine Hannover, Bünteweg 9, 30559, Hannover, Germany; 2 Institute for Animal Breeding and Genetics, University of Veterinary Medicine Hannover, Bünteweg 17p, 30559, Hannover, Germany; 3 Clinic for Small Animals, University of Veterinary Medicine Hannover, Bünteweg 9, 30559, Hannover, Germany; 4 Department of Pathology, University of Veterinary Medicine Hannover, Bünteweg 17, 30559, Hannover, Germany

**Keywords:** Ataxia, Heredity, Horse, Purkinje cells, TOE1

## Abstract

**Background:**

Cerebellar abiotrophy (CA) is a rare but significant disease in Arabian horses caused by progressive death of the Purkinje cells resulting in cerebellar ataxia characterized by a typical head tremor, jerky head movements and lack of menace response. The specific role of magnetic resonance imaging (MRI) to support clinical diagnosis has been discussed. However, as yet MR imaging has only been described in one equine CA case. The role of MR morphometry in this regard is currently unknown. Due to the hereditary nature of the disease, genetic testing can support the diagnosis of CA.

Therefore, the objective of this study was to perform MR morphometric analysis and genetic testing in four CA-affected Arabian horses and one German Riding Pony with purebred Arabian bloodlines in the third generation.

**Results:**

CA was diagnosed pathohistologically in the five affected horses (2 months - 3 years) supported by clinical signs, necropsy, and genetic testing which confirmed the *TOE1*:g.2171G>A SNP genotype A/A in all CA-affected horses.

On MR images morphometric analysis of the relative cerebellar size and relative cerebellar cerebrospinal fluid (CSF) space were compared to control images of 15 unaffected horses. It was demonstrated that in MR morphometric analyses, CA affected horses displayed a relatively smaller cerebellum compared to the entire brain mass than control animals (*P* = 0.0088). The relative cerebellar CSF space was larger in affected horses (*P* = 0.0017). Using a cut off value of 11.0% for relative cerebellar CSF space, the parameter differentiated between CA-affected horses and controls with a sensitivity of 100% and a specificity of 93.3%.

**Conclusions:**

In conclusion, morphometric MRI and genetic analysis could be helpful to support the diagnosis of CA in vivo.

## Background

Cerebellar abiotrophy (CA) is a neurological disease characterized by intrinsic spontaneous degeneration of premature neuronal cells. An inherited polymorphism is assumed to be responsible for the changes in the metabolic activity of the Purkinje neurons [1].

CA is found in Arabians and partbred Arabian horses but it has also been reported in the Gotland pony and the Oldenburg Warmblood horse [2,3]. CA is seen in several species including dogs, sheep and cattle, but it is likely that in these species, CA does not have the same aetiology as in the horse [1,4,5]. Most CA-affected horses appear to be normal at birth. Progressive degeneration of Purkinje cells causes clinical signs – in most cases between the time of birth and six months of age, however a manifestation later in life is also possible [6-10]. Affected foals often show head tremors (intention tremor), reduced or absent menace response and cerebellar ataxia. An exaggerated action of the forelegs, hyperactivity and the tendency to startle and fall are often described [8-10]. Despite distinctive clinical signs, *ante mortem* diagnosis remains suggestive and definite diagnosis needs histopathological examination [11]. Typical histopathological signs of CA are degeneration of the granular cells, disorganizations of the molecular and granular layers of the cerebellar cortex and shrunken Purkinje cells [9,10,12].

For *intra vitam* diagnosis of CA the use of MRI is not widely used or described in horses [11]. In dogs, on the other hand, several studies showed the usefulness of this diagnostic imaging technique for the diagnosis of cerebellar degenerative diseases [13-20].

Lately, efforts have been made to develop a genetic test for diagnosis of CA [8]. Since in horses a single autosomal recessive locus is assumed to be responsible for the disease, chromosomal regions have been scanned for mutations involved in CA development [8]. Recently, linkage analyses refined the map location for CA on horse chromosome (ECA) 2 at 13.05-13.19 Mb (megabases) to a conserved haplotype spanning 142 kb [8]. Complete sequencing of four annotated genes *TESK2*, *HPDL*, *TOE1* and *MUTYH* occupying approximately 49.7 kb of the 142 kb region revealed a single nucleotide polymorphism (*TOE1:*g.2171G>A SNP) within *TOE1* associated with CA in Arabian horses [8]. The aims of the current study are the assessment of subjective evaluation of brain MRI, objective MR morphometry and genetic testing regarding their value in diagnosing CA *intra vitam* in horses with histologically proven disease.

We hypothesize that MR morphometry and genetic testing can support a diagnosis of CA in Arabian horses.

## Methods

Five horses, two colts and three fillies ranging in weight from 104 to 294 kg, and aged from 2 to 36 months (median age 19 months), were examined. History of clinical signs and the patients’ pedigrees were provided by breeders or owners, respectively. Horses CA 1 to CA4 were purebred Arabian horses whereas horse CA 5 was a partbred Arabian pony.

All five horses were examined clinically and neurologically. The clinical-neurological examination included the monitoring of behavior, posture, postural reactions and gait. Cranial nerve examination, spinal reflexes and sensibility were tested.

For further diagnostic tests blood samples and cerebrospinal fluid (CSF) samples were taken under anesthesia immediately before the foals were euthanized. Thereby, 10 ml CSF were obtained under sterile conditions by an atlanto-occipital spinal tap. Hematologic and blood biochemical analysis were performed evaluating blood urea nitrogen, creatinine, lactate, glucose, bilirubin, triglycerides, sodium, calcium, potassium, chloride, aspartate aminotransferase, lactate dehydrogenase, gamma-glutamyltransferase, glutamate dehydrogenase, creatine kinase and alkaline phosphatase.

### Neuroimaging

Magnetic resonance examinations were performed under general anesthesia. For general anesthesia, horses were sedated to individual needs with xylazine (Xylazin 2%, cp-pharma, Burgdorf, Germany; 0.5-1.1 mg/kg i.v.). General anesthesia was induced with ketamine (Narketan 100 mg/mL, Vetoquinol, Ravensburg, Germany; 2.5 mg/kg i.v.) and midazolam (Midazolam 5 mg/mL, Braun, Tuttlingen, Germany; 0.06 mg/kg i.v.), and maintained with inhalation of isoflurane (Isofluran, cp-pharma, Burgdorf, Germany) in oxygen.

MRI was performed using a 3 Tesla scanner (Achieva TX, Philips Medical System, Philips GmbH, Hamburg, Germany). The heads were scanned in dorsal recumbency, four connected flexible coils were used to obtain the images. The scanning protocol included T2 Turbo spin echo sequences (T2w TSE; TR: 3000; TE: 15) in dorsal, sagittal, and transverse planes, a fluid attenuation inversion recovery (FLAIR) sequence in sagittal and transverse planes (TR: 10000; TE: 36, IR: 2600), and 3D T1 weighted pre- and post contrast sequences with a slice thickness of 4 mm. The 3D T1 sequence was reconstructed in a slice thickness of 0.9 mm. Gadoterate meglumine (Gd-DOTA; Dotarem®, Guerbet, Sulzbach, Germany) was administered at an intravenous dose of 0.02 mmol/kg to serve as a contrast agent. In one case (CA1), additionally, a T2 FFE HEMO (T2* fast field echo) transversal sequence (TR: 675 TE: 13) and diffusion sequences were performed.

**Figure 1:**
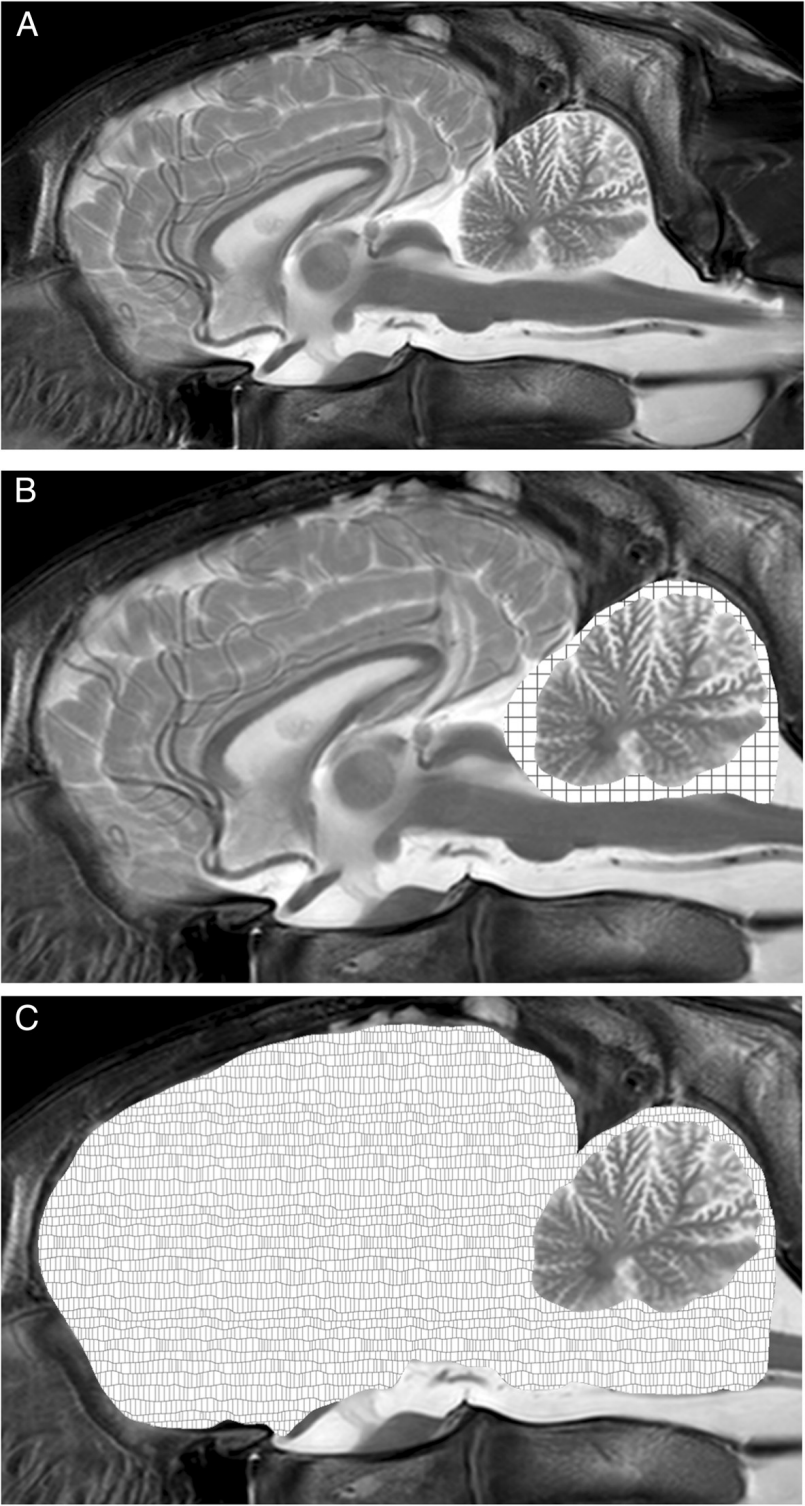
**Outline of MRI morphometric calculations on midsagittal images midsagittal T2-weighted MRI of horse CA5 (A).** The cerebellum, its surrounding CSF space and the whole brain were outlined manually and the ratio of the area of the cerebellum and the area of the cerebellum plus its surrounding CSF space (checkered pattern) was calculated (**B**). To obtain the relative cerebellar size, the area of the cerebellum was put into a ratio with the area of the whole brain (irregular pattern) (**C**).

For evaluation of the cerebellar parenchyma, sequences were assessed subjectively by two authors individually, paying special attention to the visibility of the cerebellar vermis, the amount of CSF around the cerebellum, in particular in the region of the fourth ventricle and between the folia. For morphometric analysis a midsagittal T2-weighted image (Matrix 512 × 512, voxel size of 0.3 × 0.4 × 4 mm) was chosen for each horse. The outline of the whole brain, the cerebellum and the CSF space surrounding the cerebellum was marked manually by two experienced examiners (dual-control principle) using a software suitable for analyzing veterinary diagnostic images (current release of Easyimage, VetZ, Isernhagen, Germany) (Figure 1A-C). Outline measurements were repeated twice and mean values were used for further analysis. As described elsewhere [14], relative cerebellar size and relative CSF space were calculated. Briefly, to specify the relative cerebellar size the area of the cerebellum was divided by the area of the whole brain and multiplied by 100. For assessment of the relative cerebellar CSF space, the cross sectional area of the cerebellum was subtracted from the cross sectional area of the cerebellum plus its surrounding CSF space and divided by the cross sectional area of the cerebellum plus surrounding CSF multiplied by 100.

To serve as reference values for morphometric measurements, midsagittal MR images of 15 neurologically unremarkable, adult horses’ brains were used. Identical settings as mentioned above for the MR examination of affected horses were used. Analysis was performed in one adult Arabian horse and 14 Warmbloods with a median age of 4 years (6 months to 10 years).

### Microsatellite and SNP genotyping

Genomic DNA of the affected horses (n=5) and their parents (n=10) was isolated from EDTA (ethylenediaminetetraacetic acid) blood samples (using 500 μL EDTA blood drawn from one jugular vein) by saline precipitation [21] and DNA concentration was adjusted to 10 ng/μL. Microsatellites were chosen from a marker set covering the whole equine genome (EquCab2.0^1^) at a mean distance of 112 kb [22]. Five out of twenty microsatellites in the region of 11.89-14.06 Mb were selected according to their high polymorphism information content (PIC) and heterozygosity (HET). Primer pairs were designed for the microsatellites and the *TOE1*:g.2171G>A SNP using Primer3^2^ (Additional file 1). For PCR amplification of the microsatellites and the *TOE1*:g.2171G>A SNP, reactions were assembled in 10 μL/ 30 μL total volume containing 2 μL sample DNA, 1.2 μL/ 3.1 μL incubation mix with MgCl2 (buffer; Taq Core Kit 10 (1000 U), MP Biomedicals, LLC, Germany), 0.15 μL/ 0.60 μL dNTP mix (Taq Core Kit 10 (1000 U), MP Biomedicals) at 10 mM, 0.1 μL/ 0.12 μL Taq Polymerase (5 U/ μL; Taq Core Kit 10 (1000 U), MP Biomedicals), 2.4 μL/ 6.24 μL 5× enhancer solution P (Enhancer solution, Peqlab Biotechnologie GmbH, Erlangen, Germany) and 0.03 - 0.09 μL (100 pmol/μL) 5’-IRD700 or IRD800 fluorescence labeled/ unmodified primers. Thermocycling on a PTC 200™ thermocycler (PTC 200™ thermocycler, MJ Research, Inc., Waltham, USA) was performed for both reactions using the following settings: 4 min at 94°C, followed by 36 cycles of 94°C for 30 s, 1 min at 60 °C, 30 s at 72°C and finally 10 min at 4°C. PCR products of the microsatellites were size-fractioned by gel electrophoresis on 6% polyacrylamide denaturing gels (RotiphoreseGel 40, Carl Roth) using an automated capillary sequencer (LI-COR 4200/S-2, LI-COR 4300, LI-COR Biotechnology GmbH, Bad Homburg, Germany). The data were analyzed by visual examination. Genotyping the *TOE1*:g.2171G>A SNP was performed by sequence analysis using the automated sequencer Genetic Analyzer 3500 (Genetic Analyzer 3500, Applied Biosystems by Life Technologies GmbH, Darmstadt, Germany). Haplotypes for the affected horses and their parents were reconstructed using MERLIN (version 1.1.2) [23].

### Pathology

At necropsy, the total body weight, brain weight and the weight of the isolated cerebellum were determined. Brain and spinal cord were fixed in 10% buffered formalin (pH 7.4). Randomly selected samples of cerebral cortex, corpus striatum, hippocampus, cerebellar cortex and brain stem as well as of cervical, thoracal and lumbal spinal cord were embedded in paraffin. Three μm thick sections were stained with hematoxylin and eosin (HE). Brain and spinal cord of two Arabian horses, 24 and 48 months of age and without any evidence of neurological disease, either clinical or pathological, were similarly prepared and served as controls.

### Ethical approval

All animal work has been conducted according to the national and international guidelines for animal welfare. The sampling was approved by the Lower Saxony state veterinary office Niedersächsisches Landesamt für Verbraucherschutz und Lebensmittelsicherheit, Oldenburg, Germany (registration number 33.9-42502-05-11A160).

### Statistical analysis

To evaluate the MR morphometric assessment of the relative cerebellar size and CSF space descriptive statistics of the 15 control images included median and range of values. Wilcoxon rank test was used to compare the measurements between CA affected horses and neurologically healthy horses. Significance was set at *P* < 0.05. The sensitivity and specificity of the tests were calculated using standard statistical software (graphpad prism5). The cutoff value for sensitivity and specificity calculations was set using the optimal likelihood ratio after receiver operator characteristic analysis.

## Results

At clinical examination, the vital parameters of the examined horses were within their normal range. History and clinical neurological examination of the horses were compatible with cerebellar disease in all five horses (Table 1). They were overexcited and hyperreacted to unspecified stimuli. Four horses displayed a head tremor that became more pronounced due to mental stress caused by the examination, feed intake and introduction to a new environment. Based on the neurologic examination, lesions were localized intracranially and due to reduced menace response with intact eyesight, head tremors and hypermetric ataxia, the cerebellum was identified as the site of the lesion in all horses. Complete blood cell count, blood biochemistry results and CSF analysis were within normal limits. The only deviations from normal values were hyperglycemia in all five horses, mild hyperlactatemia (1.7 mmol/L; reference 0–0.7 mmol/L) in horse CA1, and mild increase in gamma-glutamyltransferase (31 U/L; reference 0–20 U/L) in horse CA2.

**Table 1:** Patient data of 5 horses with histologically confirmed CA and comparative median values of MR morphometry of 15 control horses (controls)

	**CA1**	**CA2**	**CA3**	**CA4**	**CA5**	**Controls**
Signalment						
Gender	Filly	Filly	Colt	Colt	Filly	
Weight (kg)	212	232	244	294	104	
Clinical details						
Onset (months)	0	18	6	12	0	
Age at examination (months)	19	42	19	13	2	
Ataxia	+	-	+	+	+	
Head tremor	+	+	+	-	+	
Abnormal menace response	+	-	+	+	+	
Clinical neuroanatomic location of lesion	Cerebellum	Cerebellum	Cerebellum	Cerebellum	Cerebellum	
MR morphometry						
Relative cerebellar size (%)	15.4	19.6	17.2	19.3	16.3	20.3 (18.8-22.7)
Relative cerebellar CSF space (%)	17.4	16.3	11.8	13.5	20.8	9.4 (6.2-13.3)
Pathology						
Brain weight (g)	400	500	472	470	390	
Weight (g) (isolated cerebellum)	27	47	37	48	25	
Cerebellum: whole brain ratio (%)	6.75	9.4	7.8	10.2	6.41	
Purkinje cell degeneration	+	+	+	+	+	
Genetic testing						
CA-associated *TOE1* genotype	+	+	+	+	+	
Conserved haplotype	+	+	+	+	+	

CA - cerebellar abiotrophy.

MRI - magnetic resonance imaging.

CSF - cerebrospinal fluid.

Values in parenthesis show the range of calculated ratios for the controls.

### Molecular genetic analysis

Genotyping the *TOE1*:g.2171G>A SNP revealed that all clinically CA-affected horses had the genotype A/A while their parents showed the genotype G/A. The information about the gene flow in the pedigree on the basis of the microsatellite genotypes in the region around the *TOE1*:g.2171G>A SNP was used to reconstruct haplotypes for the individuals. The affected horses had a CA-associated homozygous haplotype 158-209-141-A-194-249 spanning the region of 11.89-14.06 Mb, while their parents were heterozygous for the CA-associated haplotype. Pedigree analysis revealed that three horses had a common ancestor while CA4 and CA5 could not be linked to that family within seven generations back (Additional file 2).

### Neuroimaging

**Figure 2:**
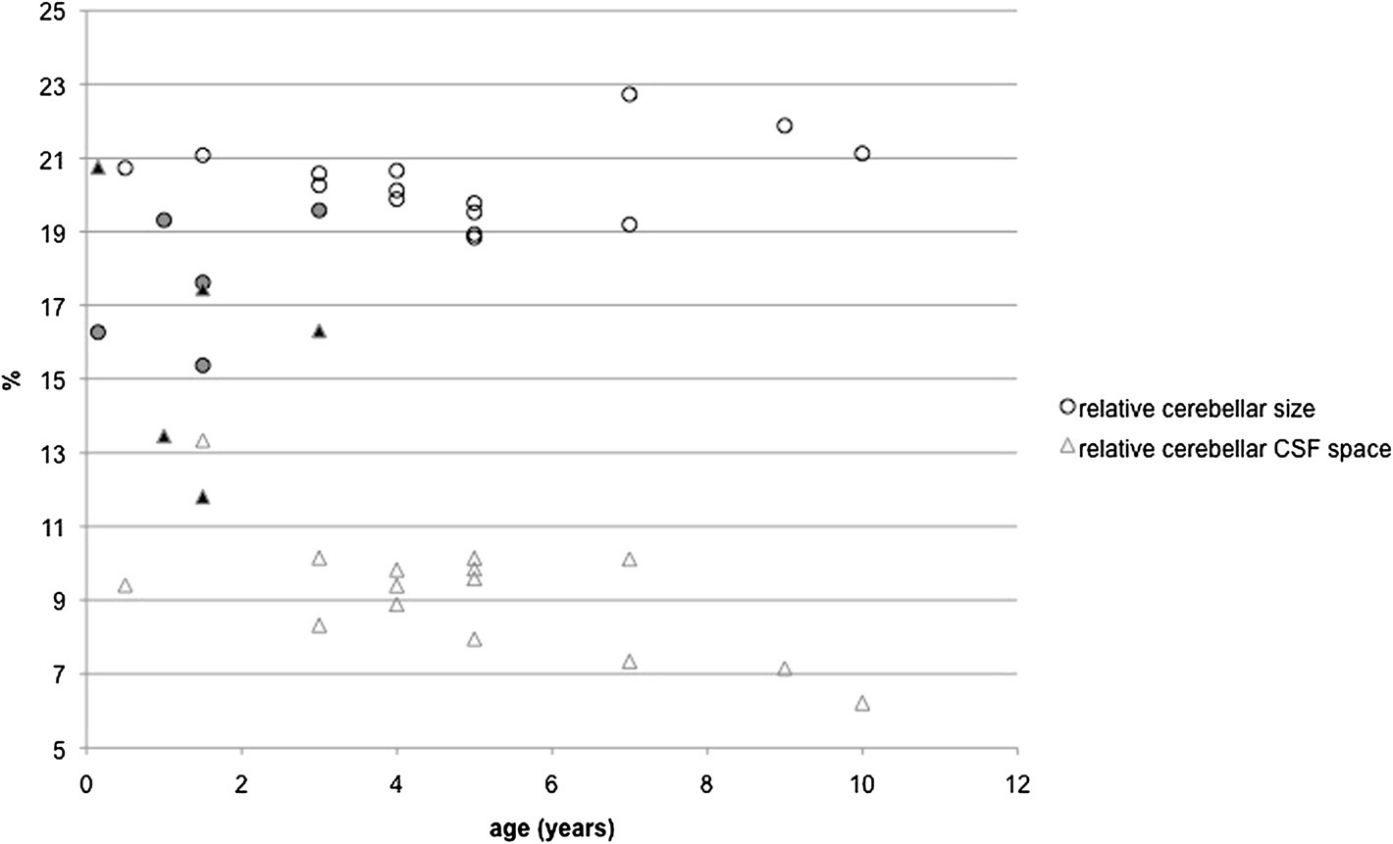
**Distribution of MR morphometric results in study population.** Filled symbols represent affected horses, unfilled symbols symbolize values obtained from 15 control MRI. The age of the horses is set against the relative cerebellar CSF space, or the relative cerebellar size.

The MR images were evaluated in sagittal, dorsal, and transverse planes. In horses CA1, CA2, and CA5 the amount of CSF in the 4^th^ ventricle and around the cerebellum, especially between the folia, subjectively seemed to be increased compared to healthy horses. In horse CA3 and CA4 this finding was subjectively not evident. Morphometric analysis on midsagittal T2 weighted images estimating the relative cerebellar size and relative CSF space revealed a significantly lower relative cerebellar size of CA-affected horses compared to healthy Warmblood horses (Wilcoxon rank test, *P* ≤ 0.0088, Table 1, Figure 2). The median relative cerebellar size of control horses (n = 15) was 20.3% (range: 18.8 – 22.7%), whereas CA-affected horses had a median relative cerebellar size of 17.6% (15.4 – 19.6%). Using a cut off value of 18.9%, MR morphometric assessment of relative cerebellar size was capable of identifying CA with a sensitivity of 60% and a specificity of 93.3%.

The median relative cerebellar CSF space of control horses was 9.4% (range: 6.2 – 13.3%). In the five CA affected horses, median cerebellar CSF space measured 16.3% (11.8 – 20.8%). This difference was significant (Wilcoxon rank test, *P* = 0.0017). Using a cut off value of 11.0%, the sensitivity of the test in identifying CA affected horses was calculated as 100% and its specificity as 93.3%.

### Pathology

**Figure 3:**
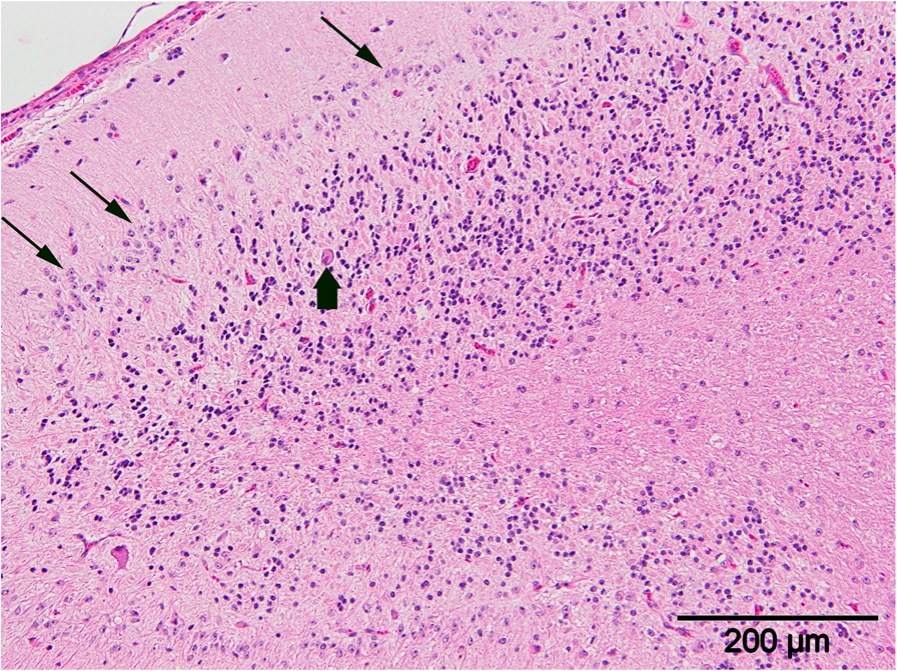
**Cerebellar folium with hypocellular granular layer and ectopic neurons (block arrow).** The Purkinje cell layer exhibits a reduced number of cells and proliferation of Bergman glia (line arrows) (hematoxilin and eosin staining, scale bars 200 μm).

On macroscopic examination the cerebellar folia appeared thinner than normal in all horses. The cerebellum to whole brain weight (cerebellum/ whole brain %) was below the lower reference value of 8% [24] in three horses (CA1, CA3, CA5) (Table 1). This corresponds well with the relative cerebellar size calculated from MRI analysis, which in these horses also showed lower values than controls. In contrast, the relative cerebellar CSF space did not mirror this cerebellar to whole brain weight ratio. Pathohistologically, the cerebellum showed a moderate to marked hypocellularity of the granular layer in all cases. Furthermore, a loss of Purkinje cells as well as ectopic Purkinje cells within the granular layer, were observed. Moreover, a proliferation of Bergmann glia next to the Purkinje cell layer was seen (Figure 3). The degree of these changes varied within different segments of the cerebellum. The sum of these changes led to the diagnosis of CA in all five cases. In addition to the above findings, CA3 showed some axonal spheroids in the cervical and lumbal spinal cord as well as a focal gliosis in the *commisura grisea* of the lumbal spinal cord. Several chromatolytic neurons were found in several basal ganglia, in the *nucleus intercalatus*, motoric *nervi hypoglossi*, *cuneatus* and *vestibularis caudalis* of the brainstem of CA2. In contrast to the affected horses, controls showed organized molecular and granular layers and intact Purkinje cells in the cerebellum. The cerebrum was unremarkable in all horses. Our findings in histopathological examinations confirmed the diagnosis of CA and was subsequently used as the gold standard in our evaluation of MRI and genetic testing as alternative diagnostic tools.

## Discussion

In the current study, we were able to demonstrate that MR brain morphometry supported the etiological and genetic diagnosis of cerebellar abiotrophy in four Arabian horses and one partbred Arabian pony foal. Pathologic and histopathologic results confirmed the diagnosis. In all cases the horses showed clinical signs of CA and the *TOE1* genotype A/A in accordance with previous reports [8]. Pathologic and histopathologic examinations revealing Purkinje cell loss and a marked hypocellularity of the granular layer verified the results. Based on those findings MR brain morphometry was able to identify CA using cut off values of 18.9% for the relative cerebellar size and 11.0% for the relative cerebellar CSF space while subjective MRI evaluation only detected rather mild changes. However, these values should not be overemphasized since there was an overlapping between normal and affected horses and the cut off value of 18.9% for the relative cerebellar size had a rather low sensitivity of 60%.

In our study, the amount of CSF around the cerebellum and between the cerebellar folia appeared to be increased in the MRI of three of the CA-affected horses. This corresponds well to the MRI findings reported in CA-affected dogs [13,14,19,20]. However, an increased visibility of the *arbor vitae* which was described in a CA-affected Arabian foal [11], was not detected in the cases studied here and in two of the affected horses subjective MRI evaluation was unremarkable.

CA is a neurologic disease affecting growing young horses with clinical signs generally beginning during the first year of life [10,11,24-30]. Although history, clinical signs and supportive laboratory data are used to make a presumptive diagnosis [31], definite diagnosis is only possible by histopathology [8,11,12]. Therefore, histopathologic evaluation was considered the gold standard in the diagnosis of CA in this study.

MRI is a non-invasive diagnostic imaging modality providing high anatomical detail and soft tissue contrast in the central nervous system. In addition, MRI of the equine head has proved to be feasible and useful in the diagnosis of neurologic diseases [32]. It has been used frequently to support the diagnosis of cerebellar degenerative diseases in dogs [13-17,19,20]. However, in the horse, only limited data are available describing MRI findings in Arabian foals suffering from CA [11].

Computer-assisted magnetic resonance imaging brain morphometry was performed to assess the relative cerebellar size and the relative CSF space surrounding the cerebellum in a midsagittal plane. Similar to the results in dogs obtained by Henke et al. [14], the morphometric analysis of both relative cerebellar size and relative cerebellar CSF space differentiated between CA-affected horses and neurologically healthy horses. However, due to the small sample size the data have to be interpreted with care. Based on sensitivity and specificity calculations, the measurement of relative cerebellar CSF space was superior to the measurement of relative cerebellar size in detecting CA-affected horses.

The MR morphometric analysis results are well supported by the measurement of the relative cerebellar weight during postmortem exam. The relative cerebellar weight (C:WB ratio) was shown to be below 8% in CA-affected horses [24]. This corresponds well to the foals in our study, showing a C:WB ratio of less than 8% in horses CA1, CA3, and CA5, in which the morphometric analysis of relative cerebellar size also showed markedly smaller values than the control horses and horses CA2 and CA4.

In this study the MR morphometric calculation of cerebellar CSF space had a stronger significance than the relative size of the cerebellum leading to a more precise detection of affected horses. The relatively low sensitivity of 60% for the relative cerebellar size could be in part explained by the small sample size of diseased horses in the current study. In American Staffordshire Terriers, relative cerebellar size was able to identify cerebellar degeneration with a sensitivity of 93% [14]. One difference in the study design was a greater sample size (14 diseased vs. 17 control dogs). The two horses with a relative cerebellar size within the control range were also the two horses that showed less clinical signs, milder histopathological changes, and a cerebellum to whole brain weight ratio within the normal range. Thus, in this study, measurement of relative cerebellar size was only able to identify CA-diseased horses correctly in advanced disease. It is open to speculation whether sensitivity might increase with a higher sample size.

Although these results are promising and give an optimistic view of the diagnostic capability of MRI for CA, it is important to note that the control MR images were obtained mainly from Warmblood horses with a median age of 4 years and not from juvenile Arabian horses. It is unclear whether these differences in age and breed have a significant influence on the obtained results. However, a study in dogs compared the percentage of the brain occupied by the cerebellum in different breeds with different skull types and found no significant differences between the breeds [19]. It seems therefore likely that the only slightly different skull shape of different horse breeds will probably not lead to measurable differences in the relative cerebellar size. Currently, there are no comparative studies on the MRI appearance and relative cerebellar size in horses of different breeds or ages.

Thus, the results suggest that MRI can not only help to confirm the diagnosis of a suspected CA case because of subjective increases in amount of CSF within and around the cerebellum, but using computer-assisted morphometric analysis in particular can provide objective criteria in support of the diagnosis.

The genetic analysis showed a highly conserved haplotype at 11.89-14.06 Mb in the region of the *TOE1* gene and the *TOE1* flanking region in all affected horses including CA4 and CA5 which were not related to the other horses. The *TOE1*:g.2171G>A SNP genotype A/A was associated with CA affliction. We assume that the haplotype surrounding the *TOE1* gene is highly conserved. This finding may suggest the existence of common founders in CA-affected Arabians many generations ago. This SNP located in exon 4 of *TOE1* results in a non-synonymous substitution of histidine for arginine. It is supposed that this substitution affects *TOE1* as a transcriptional regulator and mediator of the inhibitory growth effect of *Early Growth Response 1* (*EGR1*). The role of the SNP influencing the expression of the mutY Homolog (E. coli) (MUTYH) gene has also been discussed [8]. It can be assumed that due to the high conservation of the haplotype among the affected horses the analyzed region contains a causal mutation for CA [8,33].

## Conclusion

In conclusion, to establish a valid *ante mortem* diagnosis for CA in horses, it is desirable to provide feasible and effective diagnostic tools. Due to the genetic pathogenesis of the disease, a gene test identifying the animals at risk for developing the disease is one such tool especially suitable for early diagnosis. MR imaging together with MR brain morphometry, on the other hand, is a valid method to help to confirm the diagnosis in clinically advanced stages. On basis of clinical and genetic results MR imaging is a sensitive *intra vitam* tool to support the diagnosis of CA in manifest cases. Nonetheless, the evaluation of a larger number of horses in different states of manifestation and MR morphometric analysis of morphologically healthy brains of Arabian horses should further substantiate the results obtained in this study.

## Abbreviations

CSF: Cerebrospinal fluid; ECA: Equus caballus chromosome; EDTA: Ethylenediaminetetraacetic acid; FLAIR: Fluid attenuated inversion recovery; HE: Hematoxylin and eosin; HET: Heterozygosity; M: Molar; Mb: Megabases; mM: Millimolar; MRI: Magnetic resonance imaging; SNP: Single nucleotide polymorphism; PIC: Polymorphism information content; T2 FFE HEMO: T2* fast field echo; T2w TSE: T2 weighted turbo spin echo; TE: Echo time; TR: Repetition time; U: Units.

## Competing interests

Laboratory work was conducted at the Institute for Animal Breeding and Genetics, University of Veterinary Medicine Hannover, Foundation. Horses were housed at the Clinic for Horses, University of Veterinary Medicine Hannover, Foundation, where clinical examinations and magnetic resonance imaging were also performed. Necropsies of the horses were performed at the Institute for Pathology, University of Veterinary Medicine Hannover, Foundation. The study was not supported by a grant or otherwise. Part of the study was presented on the 2012 DVG meeting of the “Fachgruppe Pferdekrankheiten” in Hannover, Germany, March 16–17 2012 as a poster abstract. The authors state that they have no conflict of interest concerning this work.

## Authors’ contributions

JMVC, JM designed the study, carried out the experiments and data analysis, and drafted the manuscript. OD, KF, AT, AB participated in the design of the study, data interpretation and revision of the manuscript. MH, KS performed the clinical MRI examinations, VL took part in the genetic analysis and KB in the histo-pathologic examination. All authors read and approved the final manuscript.

## Supplementary Material

Additional file 1 Characteristics of the microsatellite and SNP markers used in this study. Click here for file

Additional file 2 **Pedigree of three CA-affected horses (CA1-3) and their haplotypes spanning the region at 11.89-14.06 Mb.** CA4 and CA5 showed the same haplotype but could not be linked to the pedigree. Squares: males; circles: females; solid symbols: clinically affected horses; half-filled symbols: obligate genetic carriers; unfilled symbols: unknown phenotype; black boxes: CA-associated haplotype; boxes of different patterns: haplotypes not associated with CA. All three cases have a common ancestor (**A**). Haplotypes: fragment sizes of the microsatellites and the *TOE1*:g.2171G>A SNP alleles are given according to their location on ECA2. All affected horses have a homozygous haplotype 158-209-141-A-194-249. Click here for file
