# Supplementary material for: Morphometric magnetic resonance imaging and genetic testing in cerebellar abiotrophy in Arabian horses
Source: BMC Vet Res. 2013 May 23;9:105. doi: 10.1186/1746-6148-9-105 (PMC3671216; doi:10.1186/1746-6148-9-105)
Supplement: Additional file 1 — Characteristics of the microsatellite and SNP markers used in this study. [file 1746-6148-9-105-S1.docx]

**Additional file 2.** Characteristics of the microsatellite and SNP markers used in this study

| **ECA** | **EquCab2**  **(Mb)** | **Marker** | **Acc. No.** | **Ta**  **(°C)** | **Forward / reverse primer sequence**  **(5’-3’)** | **HET (%)** | **PIC**  **(%)** | **Alleles**  **(n)** | **Allele size**  **(bp)** | **Primer amount**  **(µL)** |
| --- | --- | --- | --- | --- | --- | --- | --- | --- | --- | --- |
| 2 | 11.89 | ABGe110 | AM946989 | 60 | F: CAAGTGCCCACTGACTGATG  R: TGTTGCTGTGAATGGGACAG | 68.2 | 72.2 | 13 | 129-164 | 0.09 |
| 2 | 12.30 | ABGe1323 | FN403627 | 60 | F: TCTGTGTGGATGACTCCCAG  R: TACTCAGGAGAGGGGCAGAA | 66.7 | 71.9 | 10 | 209-229 | 0.07 |
| 2 | 12.43 | ABGe144 | FM165574 | 60 | F: CAAAAATGGCAAGATTTCATCC  R: TGCCCACTGACAGATGAATG | 64.8 | 72.4 | 14 | 115-141 | 0.03 |
| 2 | 13.07 | *TOE1*:g.2171G>A SNP | dbSNP ss # 252444770 | 60 | F: GTGCTTCCTGAGGCTAACTCC  R: ATGACGTACTCCTCCATGCAC | 17.7 | 16.9 | 2 | ─ | 0.30 |
| 2 | 13.78 | ABGe1332 | FN403696 | 60 | F: TGACGCTTCTTCCTGTAGCC  R: CAAACACGGAACACAACAGC | 65.6 | 67.5 | 9 | 180-309 | 0.05 |
| 2 | 14.06 | TKY615 | AB103833 | 60 | F: GGGCAAAGTGGTCTGAGAAG  R: CCAGACCCACCCAAAAGATA | 66.4 | 70.1 | 14 | 155-263 | 0.05 |

SNP - single nucleotide polymorphism

ECA – *Equus caballus* chromosome

Acc. No. - accession number

Ta - annealing temperature

HET - heterozygosity

PIC - polymorphism information content

bp - base pairs

Primer amount of a solution of 100 pmol/µL
