# Supplementary figures and images for: Morphometric magnetic resonance imaging and genetic testing in cerebellar abiotrophy in Arabian horses
Source: BMC Vet Res. 2013 May 23;9:105. doi: 10.1186/1746-6148-9-105 (PMC3671216; doi:10.1186/1746-6148-9-105)

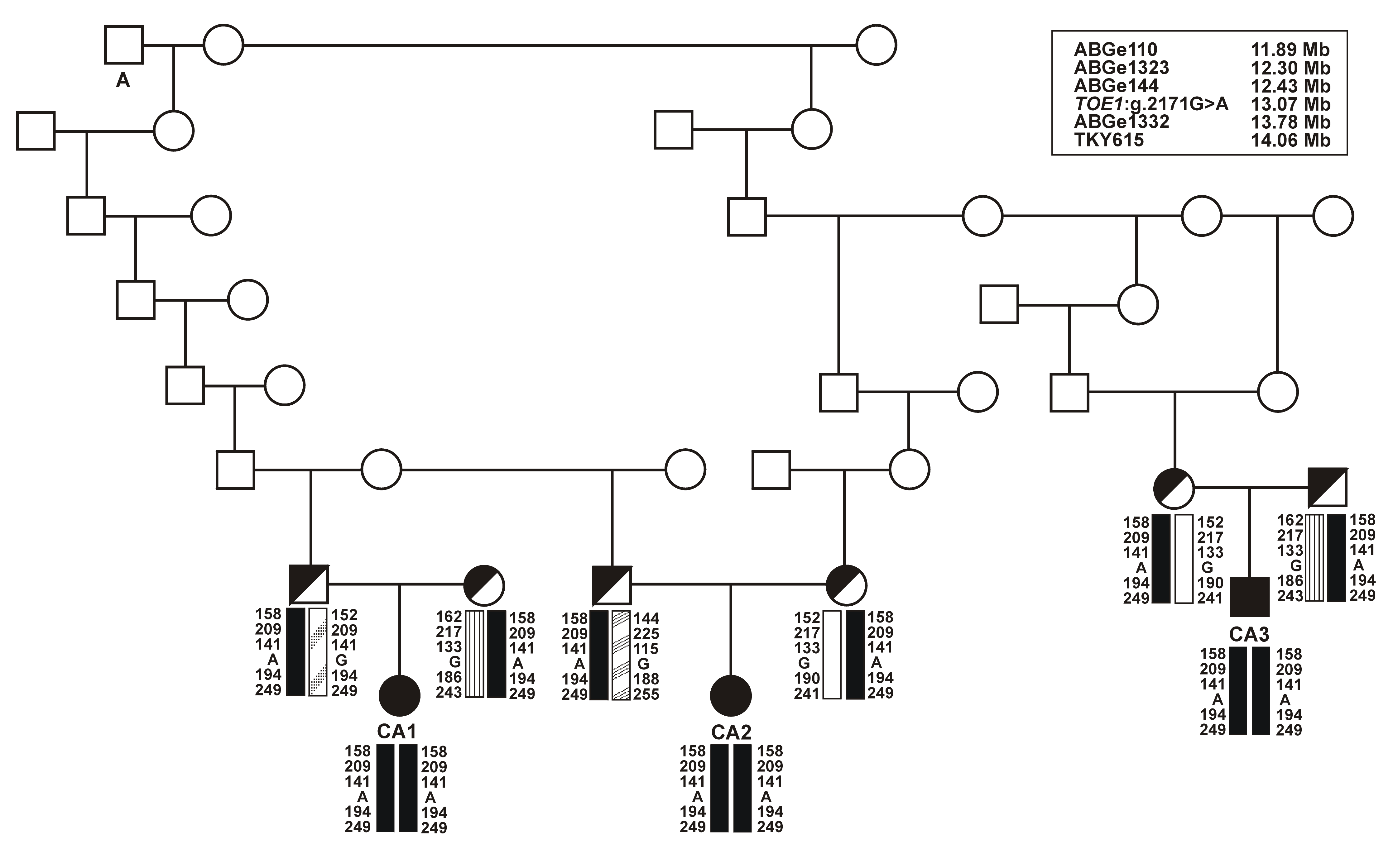

Supplement: Additional file 2 — Pedigree of three CA-affected horses (CA1-3) and their haplotypes spanning the region at 11.89-14.06 Mb. CA4 and CA5 showed the same haplotype but could not be linked to the pedigree. Squares: males; circles: females; solid symbols: clinically affected horses; half-filled symbols: obligate genetic carriers; unfilled symbols: unknown phenotype; black boxes: CA-associated haplotype; boxes of different patterns: haplotypes not associated with CA. All three cases have a common ancestor (A). Haplotypes: fragment sizes of the microsatellites and the TOE1:g.2171G>A SNP alleles are given according to their location on ECA2. All affected horses have a homozygous haplotype 158-209-141-A-194-249. [file 1746-6148-9-105-S2.tiff]
